# Modulation of olfactory sensitivity and glucose-sensing by the feeding state in obese Zucker rats

**DOI:** 10.3389/fnbeh.2014.00326

**Published:** 2014-09-17

**Authors:** Pascaline Aimé, Brigitte Palouzier-Paulignan, Rita Salem, Dolly Al Koborssy, Samuel Garcia, Claude Duchamp, Caroline Romestaing, A. Karyn Julliard

**Affiliations:** ^1^Team “Olfaction: From Coding to Memory”, Lyon Neuroscience Center, INSERM U1028-CNRS 5292- Université Lyon1Lyon, France; ^2^Laboratoire d’Ecologie des Hydrosystèmes Naturels et AnthropisésCNRS 5023, Villeurbanne, France

**Keywords:** olfactory sensitivity, obesity, olfactory bulb, glucose-sensing, GLUT4, SGLT1, extracellular glucose concentration, feeding state

## Abstract

The Zucker *fa/fa* rat has been widely used as an animal model to study obesity, since it recapitulates most of its behavioral and metabolic dysfunctions, such as hyperphagia, hyperglycemia and insulin resistance. Although it is well established that olfaction is under nutritional and hormonal influences, little is known about the impact of metabolic dysfunctions on olfactory performances and glucose-sensing in the olfactory system of the obese Zucker rat. In the present study, using a behavioral paradigm based on a conditioned olfactory aversion, we have shown that both obese and lean Zucker rats have a better olfactory sensitivity when they are fasted than when they are satiated. Interestingly, the obese Zucker rats displayed a higher olfactory sensitivity than their lean controls. By investigating the molecular mechanisms involved in glucose-sensing in the olfactory system, we demonstrated that sodium-coupled glucose transporters 1 (SGLT1) and insulin dependent glucose transporters 4 (GLUT4) are both expressed in the olfactory bulb (OB). By comparing the expression of GLUT4 and SGLT1 in OB of obese and lean Zucker rats, we found that only SGLT1 is regulated in genotype-dependent manner. Next, we used glucose oxidase biosensors to simultaneously measure *in vivo* the extracellular fluid glucose concentrations ([Gluc]_ECF_) in the OB and the cortex. Under metabolic steady state, we have determined that the OB contained twice the amount of glucose found in the cortex. In both regions, the [Gluc]_ECF_ was 2 fold higher in obese rats compared to their lean controls. Under induced dynamic glycemia conditions, insulin injection produced a greater decrease of [Gluc]_ECF_ in the OB than in the cortex. Glucose injection did not affect OB [Gluc]_ECF_ in Zucker *fa/fa* rats. In conclusion, these results emphasize the importance of glucose for the OB network function and provide strong arguments towards establishing the OB glucose-sensing as a key factor for sensory olfactory processing.

## Introduction

The Zucker “fatty” rat is a model of genetic obesity. The mutation, named fatty or *fa*, is autosomic recessive, therefore while *fa/fa* rats become obese at 3–5 weeks of age, the heterozygous *fa/+* rats remain phenotypically normal. The *fa* mutation affects the extracellular part of the leptin receptor which demonstrates a weaker affinity for leptin and an altered signal transduction (White and Martin, 1997; Yamashita et al., 1997). Leptin receptors are present in several hypothalamic nuclei playing an important role in the central regulation of energy balance (Tartaglia et al., 1995; Mercer et al., 1996; Fei et al., 1997; Shioda et al., 1998; Burguera et al., 2000). Because leptin cannot exert its anorectic action in Zucker *fa/fa* rats, these animals develop a marked hyperphagia during the first weeks of life (Vasselli et al., 1980). Consequently, the Zucker *fa/fa* rats exhibit a severe obese phenotype, highly similar to the human obese syndrome (Guerre-Millo, 1997; Beck, 2000). Zucker *fa/fa* rats display many metabolic and hormonal defects including hyperleptinemia, hyperinsulinemia, mild to moderate hyperglycemia, glucose intolerance as well as peripheral and central insulin resistance (Zucker and Antoniades, 1972; Bray, 1977; Martin et al., 1978; Ikeda et al., 1986; Pénicaud et al., 1987; Zarjevski et al., 1992; Apweiler and Freund, 1993; Guerre-Millo, 1997; Beck, 2000). As a consequence of the leptin receptor gene mutation, in the obese Zucker rats, intracerebroventricular (ICV), injections of insulin or of non-metabolized 2-deoxy-D-glucose do not change food intake in contrast these injections induce hypo- or hyper-phagia, respectively in lean Zucker rats (Ikeda et al., 1980, 1986). The absence of behavioral response to insulin and glucose ICV injections in obese Zucker rats is partly due to an impairment of hypothalamic glucose-sensing neurons. These neurons cannot adapt their firing to fluctuations of interstitial glucose concentration and cannot take part in the regulation of feeding behavior according to the metabolic needs of the organism (Rowe et al., 1996; Spanswick et al., 1997, 2000; Colombani et al., 2009). Among the molecules involved in glucose-sensing, the mRNA expression of glucokinase, a glucose metabolizing enzyme and of GLUT2, a glucose transporter is altered in the hypothalamus of *fa/fa* Zucker rats (Bogacka et al., 2004). Moreover, the regional brain glucose utilization is modified in obese Zuckers, compared to lean controls (Tsujii et al., 1988; Marfaing-Jallat et al., 1992; Doyle et al., 1993). All together these findings converge to the conclusion that, at least in brain areas well-known to be involved in the control of food intake, the neuronal glucose sensitivity is largely impaired in hyperphagic *fa/fa* Zucker rats.

Food consumption is regulated by sensory modalities among which olfaction takes an important part. In turn, olfactory sensitivity is modulated by feeding state (Aimé et al., 2007) because normal-weight Wistar rats demonstrate a better olfactory sensitivity when they are fasted than when they are satiated (Aimé et al., 2007). Moreover, ICV administrations of leptin or insulin, two anorectic hormones, decrease the olfactory sensitivity of fasted rats to the level of satiated ones (Aimé et al., 2007; Julliard et al., 2007). Conversely, central infusions of orexin A or ghrelin, two orexigenic peptides, increase the olfactory sensitivity of satiated rats to the level of fasted ones (Julliard et al., 2007; Tong et al., 2011). These converging evidences demonstrate that the olfactory bulb (OB), the first central structure responsible for the olfactory information processing, is targeted by signals (orexin A, ghrelin, leptin, insulin, NPY and CCK) involved in the regulation of energy balance (Palouzier-Paulignan et al., 2012). In addition to the hormones involved in food intake regulation, a large body of evidence indicates that the olfactory system is also sensitive to blood-borne nutrients. Two neuronal markers of glucose sensitivity, insulin-dependent glucose transporter type 4 (GLUT4) and sodium glucose co-transporter type 1 (SGLT1), are found in the olfactory system (El Messari et al., 1998). Recent patch clamp studies have shown that mitral cells change their firing rate in response to changes in glucose concentration, identifying these neurons as glucose sensors (Tucker et al., 2010, 2013) and also to changes in insulin concentration (Fadool et al., 2000; Kuczewski et al., 2014).

Given the extreme metabolic features of the obese Zucker *fa/fa* rats described above and the recent studies demonstrating a strong effect of the peripheral and central signals involved in the regulation of energy balance on olfactory processing and glucose sensitivity in the olfactory system, the present study was conducted to (i) test whether the obese Zucker *fa/fa* rats display a normal regulation of the olfactory sensitivity by the feeding states; (ii) compare the olfactory sensitivity of lean Zucker *fa/+* rats and obese Zucker *fa/fa* rats; and (iii) investigate whether the glucose concentration, GLUT4 and SGLT1 expression in the OB might be altered in the genetically obese and moderately diabetic Zucker *fa/fa* rat.

## Materials and methods

### Animals preparation

Male lean (*fa/+*; *n* = 24) and obese (*fa/fa*; *n* = 24) Zucker rats, were purchased from Charles River Laboratories. The animals were handled (5 min/day) and weighed daily. On arrival, they were 7–8 weeks old and weighed 299 ± 7.2 g (*fa/+)* and 344 ± 11.8 g (*fa/fa*). Experiments were carried out in accordance with the European Community Council Directive of November 24th, 1986 (86/609/EEC) for the care and use of laboratory animals. The Animals were housed in groups (4 rats) in plexiglas chambers at constant ambient temperature and relative humidity (22 ± 0.5°C and 50 ± 5%). They were acclimated to a 12 h light/12 h dark inverted cycle, with the light turned on at Zeitgeber time zero (ZT0). Upon arrival, the rats were given *ad libitum* access to food (chow pellets, Harlan, France) and water. 2–3 weeks prior to experimental procedure, the rats were gradually habituated to a 20 h/day food restriction (FR) schedule in which they had access to food during dark phase only, from ZT16 to ZT20. Since daily fluctuations in glycemia and insulin levels are cued by food intake (Kaul and Berdanier, 1975; Sitren and Stevenson, 1978), a single daily meal was imposed to synchronize the circadian variations of glycemia and insulin secretion among the animal cohort. Animals were handled (5 min/day) and weighed daily to assess their adaptation to FR. The daily amount of food consumed was measured for lean and obese rats in order to avoid weight loss and allow stabilization or a small gain of body weight. The daily food distribution schedule was performed by an automatic food distribution system (homemade) piloted by a computer using Matlab software.

### Behavioral procedure

**Figure 1:**
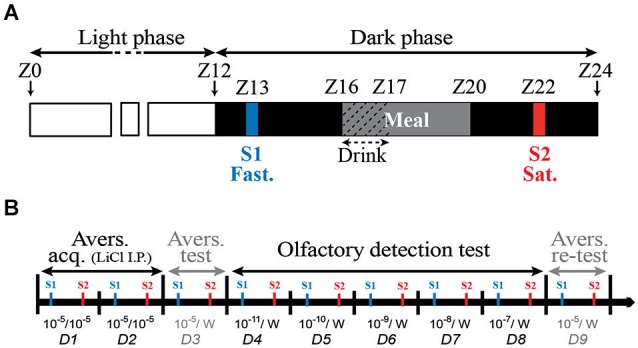
**Schematic representation of daily (A) and overall course (B) of the behavioral experiments. (A)** Lights off occurred at Zeitgeber time twelve (ZT12) and rats had access to food from ZT16 to ZT20 and to drinking water from ZT16 to ZT17. Two 5-min daily experimental sessions S1 and S2 were performed at ZT13 (before the meal: fasted) and ZT22 (2 h after food removal: satiated) respectively. **(B)** During each day of the behavioral experiment that ran from D1 to D9, all animals were successively tested in fasted (S1) and satiated (S2) states. During the aversion acquisition period (Avers. acq., from D1 to D2), rats had access to water odorized with ISO 10^−5^. ISO 10^−5^ consumption was paired with an i.p. injection of LiCl to induce a COA. On D3, corresponding to aversion test (Avers. test), the COA efficiency was tested by giving the animals the choice between water odorized with ISO 10^−5^ and pure water (10^−5^/W). During the olfactory detection test period (D4–D8), rats were offered a choice between pure water and water odorized with ISO at lower dilutions ranging from 10^−11^ to 10^−7^. On D9, (Avers. re-test) the COA stability was assessed by giving the rats the choice between ISO 10^−5^ and pure water (10^−5^/W).

The behavioral test was performed as previously described (Aimé et al., 2007, 2012; Julliard et al., 2007; Tong et al., 2011) with minor modifications, as follow. 12 Zucker *fa/+* and 12 Zucker *fa/fa* rats were submitted to the behavioral procedure. The conditioned odor aversion (COA) protocol consisted in pairing the ingestion of an odorized drink with an intraperitoneal (i.p.) injection of lithium chloride (LiCl, Sigma-Aldrich). This procedure induces a robust aversion to the odor diluted in the drinking solution. The olfactory sensitivity is then tested by measuring the aversive behavioral response to a range of lower odor concentrations. In order to induce drink intake during behavioral test, in addition to the FR, rats were placed on a 23 h water restriction schedule that started 1 week prior behavioral procedure and was maintained throughout the behavioral protocol. Animals had access to water for 1 h, during the daily food distribution. Experiments consisted of two 5-min daily sessions occurring at 9 h intervals. The first session (S1) started at the beginning of the dark phase (ZT13; after 17 h of fasting) and the second (S2) started during the dark phase, during the postprandial phase (ZT22, 2 h after the end of the meal; Figure 1A). This paradigm allowed each animal to be successively tested in two steady physiological states, fasted (S1) and satiated (S2) each day of the behavioral test. In a first period, corresponding to the habituation, rats were trained for 2 days to lick pure water at the two drinking tubes of the experimental cage (not shown). The experimental set-up allowed the recording of licking behavior using a two-tube device described elsewhere (Aimé et al., 2007, 2012; Julliard et al., 2007). During the next 2 days (Figure 1B: D1 to D2, Aversion acquisition), rats only had access to water odorized with isoamyl-acetate (ISO, Sigma-Aldrich). An ISO consumption of more than 0.5 mL was paired 15 min later, with an i.p. injection of LiCl (10 mL/kg at 0.15 M) to induce gastric malaise and establish a COA to ISO. ISO was used at the dilution of 10^−5^, for which the solution has a strong odor but has been shown to be tasteless (Slotnick et al., 1997). Thus at this dilution, the rats can only identify ISO via the olfactory pathways. Depending on individuals, 1–4 conditioning sessions (S1 and S2 for each 2 days) were required for the animal to develop a strong aversion to ISO 10^−5^. On D3 (Figure 1B: Aversion test), the COA efficiency was tested by giving the animals the choice between pure water and water odorized with ISO 10^−5^. Then the animals were submitted to the olfactory detection test (Figure 1B: D4 to D8). For this, rats were offered a choice between pure water and water odorized with ISO at lower dilutions. At the beginning of each experimental session of the olfactory detection test, the animals were placed under the odorized water tube. Rat olfactory detection for ISO was thus assessed using a forced-choice task, and not by using a simple choice task, since the thirsty rats were forced to smell the odorized tube first. This procedure was chosen to avoid the possibility that the rats, highly motivated by thirst, would go by chance to the pure water tube first, drink only water, and not sample the ISO tube. The conditioned aversion was then tested using drinking solution with ISO dilutions ranging from 10^−11^ to 10^−7^. Each given odor concentration was presented to the same rat in fasted (ZT13) and later in satiated (ZT22: *i.e*., 2 h after the food intake end) status. On D9 (Figure 1B: Aversion re-test), COA stability was assessed by giving the rats the choice between the standard ISO solution (10^−5^) and pure water.

For each concentration, an olfactory detection index was calculated. It corresponds to the proportion of the number of licks at the pure water tube normalized to the total number of licks (odorized + pure water) in the experimental device. When rats perceived ISO in the drinking solution (and consequently avoided it), they licked more pure water during the experimental session, resulting in a higher olfactory detection index. In addition, the number of licks during the first ISO consumption (first licking burst) in the experimental device was recorded. This data allowed us to accurately measure the delay necessary for the animals to be able to detect the aversive ISO diluted in the drinking solution. Data from rats starting the experimental session by drinking at the pure water tube (maximum 2 rats out of 12 per ISO dilution) was excluded from this analysis. In order to exclude a potential bias induced by the number of conditioning sessions on the strength of the COA, a Pearson correlation test was performed between the number of LiCl injections and the olfactory detection index for each animal, for each nutritional status and for each ISO concentration tested. No correlation was observed (*P* > 0.05) for both *fa/fa* and *fa/+* rats. The number of lick during the first burst, the end of the licking burst and the total number of licks at each tube during the experimental sessions were assessed and analyzed using SciPy and MySql database software (Open Source Licenses). A licking burst was defined by a train of high frequency licks (7–10 Hz) and the end of licking burst was determined when the last lick was followed by a period of inactivity lasting at least 1 s.

### Physiological measurements

To characterize the two metabolic steady states of the fasted and satiated rats, the concentrations of plasma glucose, plasma insulin, and OB insulin were measured. Peripheral blood glucose level was determined by sampling 5 μL of tail blood 1 h before and 2 h after food intake for fasted and satiated rats respectively. Glucose levels were monitored with a glucose meter (Accu-Chek®  Roche, Mannheim, Allemagne/Performa). Plasma and OB insulin levels were measured in fasted (ZT12; *n* = 6 for each genotype) or in satiated (ZT22; *n* = 6 for each genotype) state. At the end of the behavioral procedure, rats were deeply anesthetized using an i.p. injection of ketamine (Imalgene, 80 mg/kg) and xylazine (Rompun, 10 mg/kg), then rats were euthanized, and their OBs immediately frozen in liquid nitrogen. Trunk blood was collected in heparinized tubes, and the plasma fraction was separated by centrifugation at 2000 g for 5 min. Insulin was extracted from one OB per rat according to the procedure of Baskin et al. (1983) and the other OB was saved for Western blotting. To determine the influence of the yield of the insulin extraction procedure, samples with known amounts of insulin, were submitted to the same protocol. The mean extraction output was found to be ~40%. Plasma and OB insulin levels were determined using a solid-phase, two-site enzyme immunoassay following the manufacturer’s protocol (Mercodia Ultrasensitive Rat Insulin ELISA).

### Western blot analysis

One of two OB removed from rats previously used for behavioral procedure was homogenized in ice-cold homogenization buffer (Tris 5 mM, EGTA 2 mM, pH 7.4) supplemented with an anti-protease cocktail (SIGMA P8340, 10 μl per mg tissue). Western Blot analysis was performed with OB homogenates from rats euthanized either in fasted (ZT12; *n* = 4 for each genotype) or in satiated (ZT22; *n* = 4 for each genotype) state. Protein concentrations were determined by BCA assay according to the manufacturer’s recommendation (PIERCE #23225). 100 μg of proteins were loaded into 7.5% SDS-polyacrylamide gels (SDS-PAGE) and electrophoresed for 1.5 h at 110 V. Proteins were then electro-transferred for 1 h at 300 mA onto a PVDF membrane. Membranes were blocked with 5% milk in TBS containing 0.05% of Tween 20 and incubated overnight with polyclonal antibodies directed against GLUT4 (Millipore 07-1404; dilution 1:250), or SGLT1 (Santa Cruz sc-20584; dilution 1:1000). Equal loading was verified using Ponceau red stain, and by detection of the control protein β-actin (Sigma; diultion 1:8000). Membranes were washed in 0.05% Tween-PBS buffer and incubated with horseradish peroxidase-conjugated secondary antibody (dilution 1:10000). Signals were detected using the enhanced chemiluminescence detection system (Pierce #32106). Immunoblots were scanned using a desktop scanner (Epson Perfection V350) and Adobe Photoshop. Band intensities were determined using Scion Image (Scion Corporation, USA).

### Immunostaining

Animals were anesthetized (using the same protocol as described previously for ELISA and Western Blot analysis) and euthanized in the fasted (ZT12; *n* = 4 for each genotype) or in the satiated (ZT22; *n* = 4 for each genotype) state. Immunofluorescence was performed on fresh frozen brain samples by using a modification of a published method (Julliard and Hartmann, 1998). Brain cryosections were pre-incubated for 15 min with a blocking buffer containing 0.1 M phosphate buffer saline (PBS, pH = 7.4), 3% bovine serum albumin (BSA, Sigma-Aldrich) and 5% normal serum from the host species of the antibodies. The sections were then incubated overnight at 4°C with primary antibodies for SGLT-1 (R-16) (1:100; Santa Cruz Biochemicals, Santa Cruz, CA). A GLUT4 mouse antibody was used which recognizes an epitope in the cytoplasmic portion of GLUT4 [1F8] (1:100 abCam). The sections were washed with 0.1 M PBS/3% BSA and incubated for 1 h at room temperature with anti-rabbit IgG, anti-mouse IgG, or anti-goat secondary antisera coupled to Alexa 488 (1:100), Cy3 (1:200; Jackson Immunoresearch), or Cy5 (1/100; Jackson Immunoresearch) respectively. After the final wash with PBS, slides were mounted with Vectashield mounting medium containing DAPI for nuclear staining (Vector Laboratories). Images were acquired using a Zeiss Apotome epifluorescence microscope equipped with a digital camera and Axiovision software.

### Bioprobe measurements of extracellular glucose

#### Surgical procedure

**Figure 2:**
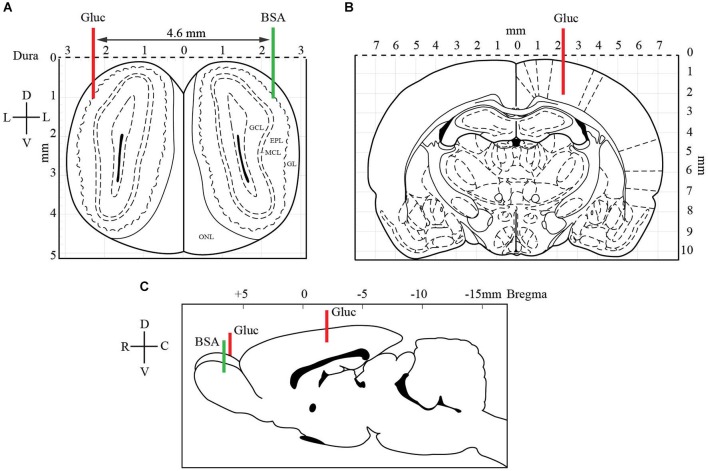
**Localization of glucose-oxidase biosensors (red) and control BSA sensor (green) electrodes in OB (A, C) and somatosensory cortex (B, C)**. At the OB level **(A)**, two electrodes were implanted symmetrically (coordinates: AP +6.5 mm from Bregma, M/L −2.3 mm and D/V −1.0 mm from dura). A glucose-oxidase biosensor (Gluc, red line) was positioned in one bulb and a control BSA sensor (BSA, green line) in the other bulb. The latter was set measure nonspecific variations in oxidation current. In the somatosensory cortex **(B)**, contralateral to the OB glucose sensor, the second glucose-oxidase biosensor was inserted (coordinates: AP −3.5 mm from Bregma, M/L +2.3 and D/V −2.3 mm from dura). The coronal drawings are from the Paxinos and Watson (2007) atlas, plates 1 and 27 (EPL: external plexiform layer, GCL: granular cell layer, GL: glomerular layer, MCL: mitral cell layer).

Eight Zucker *fa/+* and eight Zucker *fa/fa* rats were used during measurements of extracellular glucose in the brain. Rats in the fasted (ZT12, *n* = 4 for each genotype) or satiated (ZT22, *n* = 4 for each genotype) state were anesthetized with urethane (1.5 mg/kg, i.p.). The anesthetized animals were placed in a stereotaxic apparatus and kept on a heating pad, and additional doses of urethane were supplied as needed. The surgical procedure consisted of drilling three burr holes into the skull to expose the lateral region of each OB and the somatosensory cortex as a control brain area. The first glucose-oxidase biosensor was implanted into the lateral part of one OB, within or close to the glomerular layer (GL; coordinates: AP +6.5 mm from Bregma, M/L −2.3 mm and D/V −1.0 mm from dura, Figure 2). The second glucose-oxidase biosensor was inserted in the controlateral somatosensory cortex (coordinates: AP −3.5 mm from Bregma, M/L +2.3 and D/V −2.3 mm from dura). A control BSA sensor was implanted in the other OB to measure nonspecific variations in oxidation current (coordinates similar to glucose biosensors implanted in OB, Figure 2). A reference electrode (Ag/AgCl) was placed into neck muscles during *in vivo* recordings.

#### Preparation of electrochemical sensors

The glucose biosensor uses glucose oxidase and amperometric detection of hydrogen peroxide (Vasylieva et al., 2011). The tip of the probe was coated with glucose oxidase only on 100–150 μm in length (Vasylieva et al., 2011) to target mainly the GL, which thickness is ranging from 100 to 300 μm. Glucose oxidase metabolizes glucose in the extracellular fluid. An oxidation current is thus generated and measured using Neurolabscope software. Each biosensor was connected to a potentiostat, which sent readings of the current generated by glucose in extracellular fluid to a computer. Glucose biosensors have been shown previously to have a range of 0–10 mM with *in vitro* sensitivity of 1.6 ± 0.4 nA/mM (mean ± SEM). To confirm the accuracy of the biosensors, prior to implantation and immediately following testing, biosensor probe were placed in 0.1 M PBS, connected to the potentiostat, and readings were allowed to stabilize (generally stable within 15–30 min).

#### Selectivity and calibration of biosensors

Before the experiments all biosensors were tested for detection of serotonin (5-HT, 20 μM in PBS; Sigma) and H_2_O_2_ (1 μM in PBS). Only electrodes exhibiting less than 1.2 μA.mM^−1^ cm^−2^ response for 5-HT were included in the study. *In vitro* calibrations were performed in standard PBS (0.01 M, pH 7.4) and solutions were maintained at a temperature of 36.5°C, comparable to the brain of an anesthetized rat (Zhu et al., 2004). The reference electrode (Ag/AgCl) was placed directly in the solution. After a stable baseline reading, the glucose sensors were calibrated using glucose solutions at different concentrations (0; 0.5; 1; 1.25 and 1.5 mM) to establish the nA/mM ratio. The applied voltage for amperometric studies was +500 mV. Biosensors were calibrated before and after each real-time *in vivo* experiment to ensure the sensitivity remained stable. Quantitative assessments of brain glucose concentrations were obtained by subtracting the non-specific current of the control biosensor (BSA) from the output of the glucose biosensors.

#### Real-time measurements *in vivo*

To compare glucose level variations in the OB and the cortex to the peripheral glucose concentrations, 5 μl blood samples were collected from the femoral artery at the beginning of the surgery and then every 10 min thereafter. Glucose readings were performed with a glucose meter (Accu-Chek®  Roche, Mannheim, Allemagne/Performa).

Measurements of extracellular glucose in rat brains started 1 h after the implantation of the electrodes, to allow restoration of the blood-brain barrier. Fasted rats (*n* = 4 for each genotype) were anesthetized before food intake, and satiated rats (*n* = 4 for each genotype) after food intake.

Recordings started once the electrodes were implanted, and lasted for approximately 3 h. Currents obtained after the signal stabilization corresponded to the initial steady state of the animal, *i.e*., fasted or satiated. To evaluate possible fluctuations of central glucose level during dynamic glycemia conditions, an i.p. injection of glucose (Lavoisier, Paris, France 30%; i.p. 3 g/kg) to the fasted rats, or a subcutaneous injection of insulin (Sigma, Saint Quentin-Fallavier, France; 7.5 U/mL; subcutaneous, 25 U/kg) to the satiated rats, were given to measure the effects of acute modifications in peripheral glucose levels on central structures (OB and cortex). 1 h later, rats in the induced hyperglycemic state were injected with insulin and rats in the induced hypoglycemic state were injected with glucose. In 1 of 9 satiated rats glucose was monitored in the OB alone, and three rats received only insulin injection. In 1 of 8 fasted rats, glucose was monitored only in the OB, and three rats received only glucose injection. When recordings ended, rats were euthanized using sodium pentobarbital (i.p. 3 g/kg).

### Statistical analysis

Data are shown as mean values ± SEM. For olfactory detection all percentage measures were transformed using the arcsine square root transformation to normalize the data and stabilize variance (Sokal and Rohlf, 1981). For behavioral data, Western Blot data, and extracellular glucose measurements, statistical analysis were performed by either a Student’s *t*-test or one- or two-way repeated-measures ANOVA depending on the data set. A Student-Newman-Keuls (SNK) *post hoc* test was used to complete the analysis when appropriate (Statview software). Physiological parameters (glycemia, plasma and OB insulin level) were analyzed using a non-parametric Mann-Whitney test, or Wilcoxon test when data were paired (Statview Software).

## Results

### Effect of food restriction on body weight of lean and obese zucker rats

**Figure 3:**
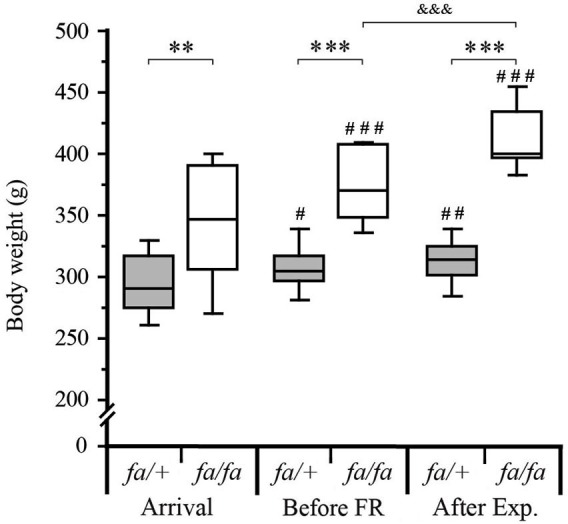
**Monitoring of body weight of *fa/+* and *fa/fa* Zucker rats during the experiments**. On their arrival, the body weight of the *fa/+* and *fa/fa* Zucker rats (7–8 weeks old), was already significantly different (299 ± 7.2 g *vs*. 344 ± 11.8 g lean *vs*. obese respectively; ** *P* < 0.001). Between their arrival and before food restriction (FR, 10–11 weeks old) the body weight of the two Zucker rats genotype had increased (299 ± 7.2 g to 306 ± 5.7 g for lean; ^#^ *P* < 0.05 and 344 ± 11.8 g to 372.9 ± 7.4 g for obese; ^###^ *P* < 0.001) and the body weight difference between lean and obese rats was more pronounced (*** *P* < 0.0001). After the experimental procedure (After Exp) corresponding to 2 weeks of FR, the body weight of *fa/+* rats stayed significantly different compared to their body weight measured at their arrival (^##^ *P* < 0.001) but it was not significantly different compared to those measured before FR (306 ± 5.7 g *vs*. 312.3 ± 4.4 g; *P* = 0.1). The *fa/fa* rats body weight had continued to increase (372.9 ± 7.4 g before FR *vs*. 412 ± 7.1 g after Exp.; ^&&&^ *P* < 0.0001). Values are shown as medians ± interquartile range, ^#^ compared to the body weight at the arrival of each genotype group the statistical analyses were performed using paired *t*-test, *n* = 16 per genotype.

Before any other experiment, *fa/+* and *fa/fa* Zucker rats were gradually habituated to a 20 h/day FR schedule (Figure 1A). In order to determine the effect of FR schedule on weight gain, body weight of the rats was measured, on their arrival, just before the FR beginning and after 3–4 weeks of FR corresponding to the end of the experimental procedures (Figure 3). On their arrival, the rats were 7–8 weeks old and the body weight of the two genotypes was already significantly different (299 ± 7.2 g *vs*. 344 ± 11.8 g in *fa/+* *vs*. *fa/fa* respectively; *P* < 0.001 Mann Whitney test). At the end of the experimental procedures, the difference in the body weight between *fa/+* and *fa/fa* and rats was larger (312 ± 7.1 g *vs*. 412 ± 7.1 g in *fa/+ vs*. *fa/fa* respectively; *P* < 0.0001 Mann Whitney test). While the *fa/+* rats’ body weight remained stable during the FR, (306 ± 5.7 g *vs*. 312.3 ± 4.4 g before FR *vs*. after the experimental procedure, *P* = 0.1, paired *t*-test) the *fa/fa* rats’ body weight had continued to increase (372.9 ± 7.4 g *vs*. 412 ± 7.1 g before FR *vs*. after the experimental procedure, *P* < 0.0001 paired *t*-test).

### Effect of feeding state on glycemia, insulinemia, and ob insulin levels in lean and obese zucker rats

In order to characterize the physiological hallmarks of the two genotypes of Zucker rats and to evaluate the physiological effects of the feeding state, glycemia, as well as plasma and OB insulin levels were measured in both 20 h fasted and satiated *fa/+* and *fa/fa* Zucker rats (Table 1). A two-way ANOVA with feeding state and genotype as factors revealed a significant effect of these two factors on all three measurements; glycemia (*F*_(1,20)_ = 8.4, *P* < 0.01 and *F*_(1,20)_ = 5.0, *P* < 0.05 respectively), plasma (*F*_(1,20)_ = 14.4, *P* < 0.005 and *F*_(1,20)_ = 247.3, *P* < 0.0001 respectively) and OB insulin levels (*F*_(1,20)_ = 38.7, *P* < 0.0001 and *F*_(1,20)_ = 320.7, *P* < 0.0001 respectively). For each genotype, the three measurements were significantly higher in satiated than in fasted rats (Table 1: Mann-Whitney tests).

**Table 1:** **The effects of feeding state on glycemia, plasmatic and OB insulin levels in lean *fa*/+ and obese *fa/fa* Zucker rats**.

	Zucker genotype	Fast. *n* = 6	Sat. *n* = 6	Mann-Whitney Sat. vs. Fast.	Mann-Whitney *fa/+ vs. fa/fa*	
					Fast.	Sat.
Glycemia (mM)	*fa/+*	6.15 ± 0.2	7.0 ± 0.4	P < 0.05	ns	P < 0.05
	*fa/fa*	6.51 ± 0.3	10.67 ± 1.7	P < 0.005
Plasmatic insulin (μg/L)	*fa/+*	1.04 ± 0.04	2.52 ± 0.02	P < 0.005	P < 0.005	P < 0.005
	*fa/fa*	10.39 ± 0.5	13.90 ± 0.2	P < 0.05
OB insulin (ng/g)	*fa/+*	0.16 ± 0.01	0.28 ± 0.1	P < 0.005	P < 0.005	P < 0.005
	*fa/fa*	0.62 ± 0.01	0.86 ± 0.02	P < 0.005

*For each genotype, glycemia, OB and plasma insulin levels were statistically higher in satiated compared to fasted animals. For each feeding state (fasted; Fast. and satiated; Sat.) levels of these three types of measure were significantly higher in *fa/fa* rats compared to their *fa*/+ counterparts, except for glycemia of fasted rats (Mann-Whitney test, *n* = 6/feeding state/genotype Zucker, ns: *P* > 0.05)*.

By comparing the two genotypes, obese *fa/fa* rats were moderately hyperglycemic (only in satiated state), compared to lean *fa/+* rats and their insulin levels (in plasma and in OB) were higher than in *fa/+* regardless of the feeding state (Table 1: Mann-Whitney tests). The difference in insulin concentration between the two rat strains was smaller in the OB (3–3.87 fold more concentrated in satiated and fasted states, respectively) than in the plasma (5.5–10 fold more concentrated in satiated and fasted states, respectively).

### Effect of feeding state and of genotype on olfactory detection

**Figure 4:**
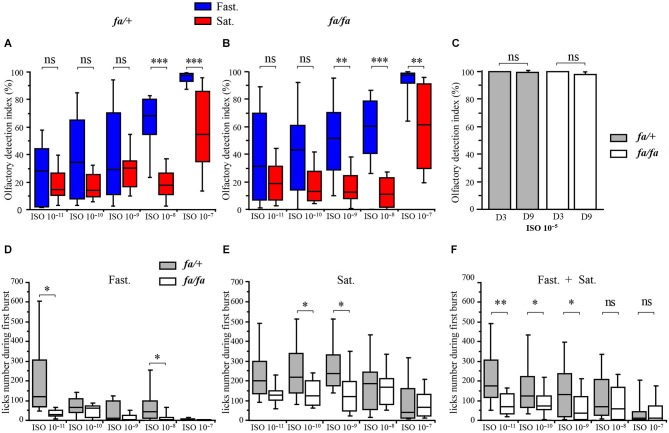
** Modulation of olfactory abilities of *fa/+* and *fa/fa* Zucker rats by feeding state**. Each animal was successively tested in fasted (Fast., in blue) and satiated (Sat., in red) states. **(A, B, D, E, F)** Values are shown as medians ± interquartile range, *n* = 6 per group (satiated and fasted rats); per rat genotype (*fa/+* and *fa/fa* rats); per ISO dilution. **(A, B)** Box graphs showing the olfactory detection index corresponding to the percentage of licks at the pure water tube normalized to the total number of licks in the experimental cage when rats were given the choice between pure water and odorized water at ISO 10^−11^, ISO 10^−10^, ISO 10^−9^, ISO 10^−8^, ISO 10^−7^. The mean olfactory detection indexes were significantly higher for fasted *fa/+* rats compared to satiated *fa/+* rats for ISO 10^−8^ and ISO 10^−7^. The mean olfactory detection indexes were significantly higher for fasted *fa/fa* rats compared to satiated *fa/fa* rats for ISO 10^−9^, ISO 10^−8^ and ISO 10^−7^. **(C)** Aversion test and aversion re-test. Bar graphs showing the olfactory detection indexes of *fa/+* (gray bars) and *fa/fa* (white bars) rats for the aversion test (Avers. Test, D3) and re-test (Avers. re-test D9). The olfactory detection indexes for *fa/+* and *fa/fa* rats were not significantly different and close to 100% before (at day 3) and after (at day 9) the olfactory detection test.** (D–F)** Box graphs showing the number of licks during the first ISO consumption in the experimental device. **(D)** In fasted state (Fast.), the mean number of licks during the first ISO consumption was significantly lower for *fa/fa* rats compared to *fa/+* rats for ISO 10^−11^ and ISO 10^−8^. **(E)** In satiated state (Sat.), the mean number of licks during the first ISO consumption was significantly lower in *fa/fa* rats compared to *fa/+*rats for ISO 10^−11^ and ISO 10^−9^. **(F)** The mean number of licks during the first ISO consumption was significantly lower for *fa/fa* rats compared to *fa/+* rats for ISO 10^−11^, ISO 10^−10^ and ISO 10^−9^ regardless to feeding state.

In order to compare the olfactory detection abilities of *fa/+* and *fa/fa* rats in fasted and satiated states, a behavioral test based on COA was performed and olfactory detection indexes were measured for ISO dilutions ranging from 10^−11^ to 10^−7^ (Figures 4A,B). During the olfactory detection test (Figure 1B, from day 4 to day 8, D4 to D8), a two-way ANOVA with feeding state and odor as factors revealed significant effects of feeding state (*fa/+* *F*_(1,88)_ = 15.8, *P* < 0.01; *fa/fa F*_(1,88)_ = 30.4, *P* < 0.0001) and odor (*fa/+* *F*_(4,88)_ = 27.1, *P* < 0.0001; *fa/fa* *F*_(4,88)_ = 15.8, *P* < 0.0001) on olfactory detection indexes. *Post hoc* analyses (paired *t*-test) revealed that *fa/+* rats have higher olfactory detection indexes in the fasted state compared to the satiated state, for ISO 10^−8^ (fasted: 62.6 ± 6.8%, satiated: 19.5 ± 3.9%, *P* < 0.0001) and ISO 10^−7^ (fasted: 95.3 ± 1.7%, satiated: 55.6 ± 9.0%, *P* < 0.0005). This effect was even more pronounced in *fa/fa* rats, which have higher olfactory detection indexes in the fasted state compared to the satiated state for a concentration as low as ISO 10^−9^ (fasted: 51.3 ± 9.0%; satiated: 17.0 ± 4.0%, *P* < 0.01) but also for ISO 10^−8^ (fasted: 58.2 ± 7.4%, satiated: 12.9 ± 3.2%, *P* < 0.0001) and ISO 10^−7^ (fasted: 89.8 ± 5.6%, satiated: 61.2 ± 9.2%, *P* < 0.005).

To ensure that the COA was robust and maintained throughout the olfactory detection test, the olfactory detection indexes were measured at ISO 10^−5^ for all the animals, before (Figure 1B, Aversion test, on D3) and after (Aversion re-test, on D9) the olfactory detection test. During the aversion test (D3), carried out after the conditioning, the animals licked almost exclusively from the pure-water tube, as demonstrated by olfactory indexes close to 100% (Figure 4C, fasted *fa/+*: 99.97 ± 0.03%, satiated *fa/+*: 99.98 ± 0.02%, fasted *fa/fa*: 99.85 ± 0.15%, satiated *fa/fa*: 99.80 ± 0.10%) showing that the COA was well established at the beginning of the olfactory detection test. On the last day of the behavioral experiment (Aversion re-test, D9), all the animals drank, again, almost exclusively at the pure-water tube (fasted *fa/+*: 99.90 ± 0.06%, satiated *fa/+*: 99.38 ± 0.23%, fasted *fa/fa*: 98.01 ± 1.80%, satiated *fa/fa*: 98.05 ± 0.90%), indicating that COA was maintained throughout the behavioral experiment.

In order to further compare the olfactory detection abilities of *fa/+* and *fa/fa* rats, the number of licks were measured during the first consumption of each given ISO concentration in the experimental device (Figures 4D–F). This measurement shows the number of licks that were necessary for the animal to detect the aversive ISO odor diluted in the drinking solution. One should expect that the better the animal can detect the ISO, the lower the number of licks during the first consumption would be. A three-way ANOVA with genotype, feeding state and odor as factors revealed significant effects of these three factors on the number of licks during the first ISO consumption (genotype: *F*_(1,132)_ = 4.3, *P* < 0.05, feeding state: *F*_(1,132)_ = 15.5, *P* < 0.0005, odor: *F*_(4,132)_ = 7.1, *P* < 0.0001). Fasted animals required fewer licks than satiated animals to detect and leave the aversive solution (*post hoc* analysis SNK), further demonstrating that animals have better olfactory sensitivity when fasted than satiated. In fasted states (Figure 4D), *fa/fa* rats required fewer licks than *fa/+* rats to detect and leave the aversive solution at ISO 10^−11^ (*fa/fa*: 33.89 ± 7.6, *fa/+*: 220.3 ± 78.0, *P* < 0.05 *t*-test), and at ISO 10^−8^ (*fa/fa* 15.67.5 ± 7.3, *fa/+*: 80.17 ± 29.2, *P* < 0.05 *t*-test). In satiated state (Figure 4E) *fa/fa* leaved the aversive solution faster than *fa/+* rats at ISO 10^−10^ (*fa/fa*: 138.25 ± 21.2, *fa/+*: 247.67 ± 44.3, *P* < 0.05 *t*-test) and also at ISO 10^−9^* (fa/fa*: 142.41 ± 35.2, *fa/+*: 275.33 ± 40.0, *P* < 0.05 *t*-test). In addition when data obtained in fasted and in satiated states were pooled (Figure 4F), the lick number during the first burst was significantly lower for *fa/fa* rats compared to their lean counterparts at ISO 10^−11^, ISO 10^−8^ and ISO 10^−9^ (*P* < 0.001, *P* < 0.05, *P* < 0.05 respectively, *t*-test). Neither genotype (*fa/+ vs. fa/fa* for each feeding state: *t*-test) nor feeding state (fasted vs. satiated for each genotype: paired *t*-test) effect was observed on the lick number of the first burst at the pure water tube during the habituation period observed (*P* > 0.05, fasted rats: *fa/+* 76.16 ± 21.5, *fa/fa* 55.16 ± 6.11; satiated rats: *fa/+* 110.8 ± 17.1, *fa/fa* 78.25 ± 11.1). Altogether, these results indicated that *fa/fa* rats have a better olfactory sensitivity than *fa/+* rats.

### Effects of feeding state on expression of GLUT4 and SGLT1 in OB of the two genotypes

**Figure 5:**
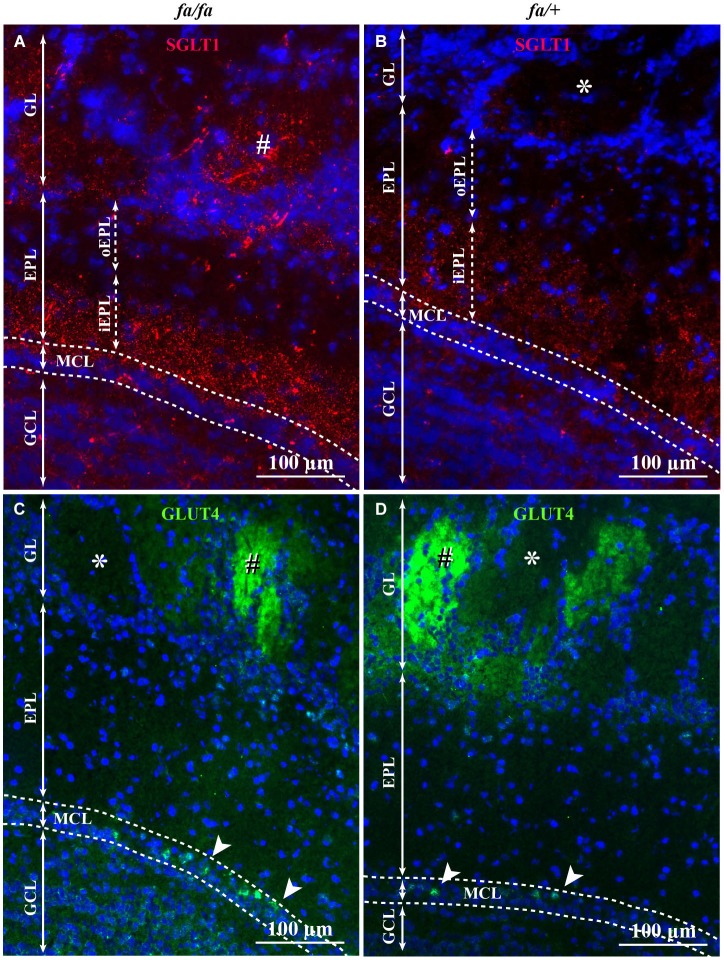
**SGLT1 and GLUT4 localization in OB layers of *fa/+* and *fa/fa* Zucker rats**. Representative SGLT1 **(A,B)** and GLUT4 **(C,D)** immunolocalization among OB layers of *fa/fa* **(A,C)** and *fa/+* **(B,D)** Zucker rats merged with the nuclear marker DAPI. In *fa/fa* **(A)** and *fa/+* **(B)** Zucker rats, SGLT1 was mainly found in the inner part of the EPL (iEPL) and the overall expression of SGLT1 is stronger in Zucker *fa/fa* rats. The outer part (oEPL) was unlabeled. In the GL, some (#) but not all glomeruli express SGLT1. The number of SGLT1-positive glomeruli is higher in Zucker *fa/fa* rats compared to Zucker *fa/+* rats. Very slight SGLT1 immunostaining was also found in the GCL. In *fa/fa* **(C)** and *fa/+* **(D)** Zucker rats, no difference of immunolocalization of GLUT4 was observed. GLUT4 was located in the glomerular layer. Glomeruli showed different level of GLUT4 expression (strongly labeled: #, not labeled: *). Some mitral cells (arrowhead) were intensely immunostained (EPL: external plexiform layer, GCL: granular cell layer, GL: glomerular layer, MCL: mitral cell layer).

In order to analyze the localization of SGLT1 and GLUT4 within the different OB layers in Zucker *fa/fa* and *fa/+* rats, an immunofluorescence experiment was performed. SGLT1 and GLUT4 were found in different layers of the OB. In both lean and obese Zucker rats, immunostaining of SGLT1, a transporter of non-metabolized glucose, revealed a layer-specific pattern (Figures 5A,B). The highest staining was observed in the inner part of EPL (iEPL) with a stronger expression in *fa/fa* rats (Figure 5A). In the two genotypes, the outer part of the EPL (oEPL) was unlabeled (Figures 5A,B). SGLT1 immunostaining was also detected in some glomeruli more particularly in *fa/fa* Zucker rats. In both lean and obese Zucker rats, the highest level of GLUT4 immunostaining was detected in the glomerular layer (GL) and in the mitral cell layer (MCL; Figures 5C,D) while the nerve layer appeared unstained. Within the glomerular neuropil, GLUT4 staining varied from strongly to unlabeled glomeruli. The pictures shown in the manuscript are representative of SGLT1 and GLUT4 immunostaining in the OB of both rat genotypes and were consistent across rats within each group. We could not see any difference in the localization of GLUT4 and SGLT1 in fasted rats compared to satiated rats, for both genotypes (data not shown).

**Figure 6:**
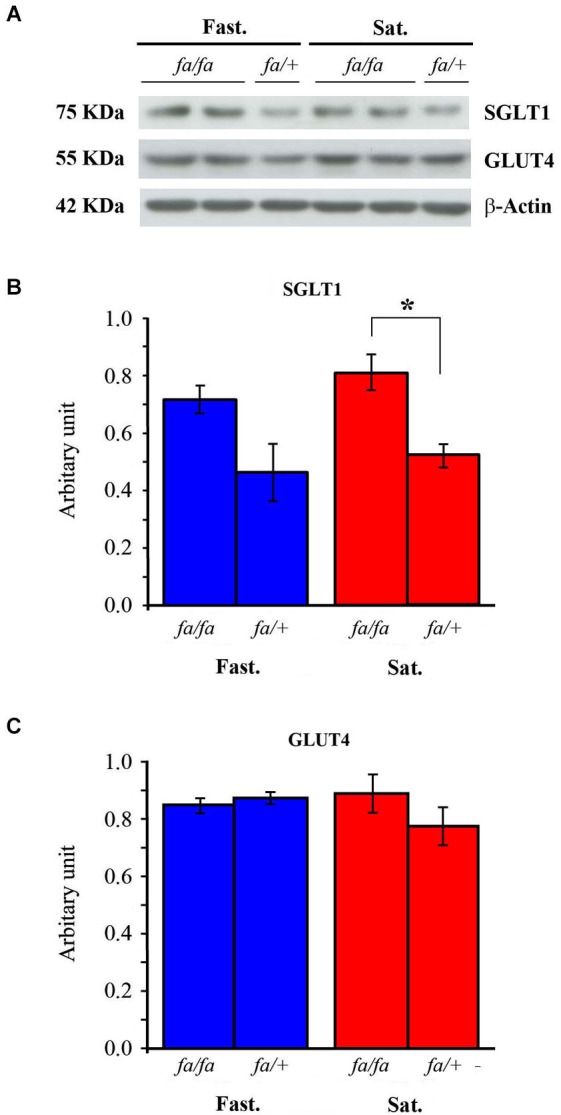
**Western blot analysis of SGLT1 and GLUT4 in OB homogenates from fasted (Fast.) or satiated (Sat.) *fa/fa* and *fa/+* Zucker rats. (A)** A representative blot****. The intensity of each band was measured by densitometry and normalized to the housekeeping protein actin, as a loading control. **(B)** SGLT1 levels were significantly greater in satiated *fa/fa* rats compared to satiated *fa/+* rats. **(C)** GLUT4 levels were not significantly different significantly regardless of the rat genotype and the feeding state. * *P* < 0.05, mean ± S.E.M., *n* = 4/feeding state/genotype.

The effects of feeding state and genotype on GLUT4 and SGLT1 protein levels in the OB were analyzed by Western blotting (Figure 6). Total tissue GLUT4 and SGLT1 were analyzed using the total protein recovery from the OBs of four satiated and four fasted rats of each genotype (Figure 6A). A two-way ANOVA on SGLT1 protein levels (Figure 6B), with feeding state and genotype as factors, revealed no effect of feeding state, but a significant effect of genotype (*F*_(1,12)_ = 14.87, *P* < 0.005). *Post hoc* analyses (SNK) of these data confirmed a higher expression of SGLT1 in *fa/fa* Zucker rats compared to *fa/+* rats. This higher expression is observed in the satiated state only, as revealed by Mann-Whitney test (*P* < 0.05). Neither feeding state nor genotype had an effect on GLUT4 levels (Figure 6C) (*F*_(1,12)_ = 0.54, *P* = 0.47 and *F*_(1,12)_ = 1.146, *P* = 0.3, respectively). Together these results indicated that SGLT1 protein levels were significantly increased in the OB of Zucker *fa/fa* rats compared to *fa/+* rats.

### Real time *in vivo* Monitoring of extracellular fluid glucose in ob and cortex of lean and obese zucker rats

#### During steady states

**Figure 7:**
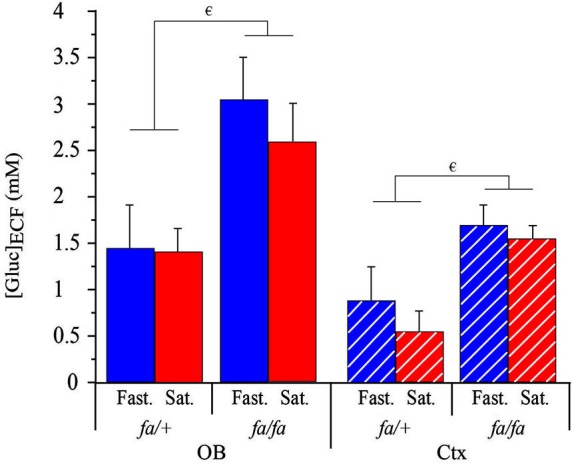
**Genotype effect on the [Gluc]_ECF_ at steady state in OB and cortex**. [Gluc]_ECF_ in OB (full bars) and in cortex (Ctx, hatched bars) of lean *fa/+* (left) and obese *fa/fa* (right) in fasted (Fast., blue) and satiated (Sat., red) sates. A three-way ANOVA with brain areas (OB and cortex), feeding state and genotype as factors showed a significant effect of brain areas (not shown) and genotype (not shown). Both in OB and Ctx, [Gluc]_ECF_ were higher in *fa/fa* compared to *fa/+* (Mann Whitney tests, ^€^ *P* < 0.05 for each area brain). For each genotype, [Gluc]_ECF_ was significantly higher in OB compared to Ctx (not shown). Values are expressed as the mean ± SEM, *n* = 4/feeding state/genotype.

Extracellular fluid glucose concentration ([Gluc]_ECF_) was measured simultaneously in the OB glomerular layer and the somatosensory cortex of lean or obese Zucker rats, in fasted or satiated state. 2 weeks prior to the experimental study, animals were habituated to a 2 h feeding/22 h starvation schedule. At the beginning of the experiment, the metabolic status of the rats was considered to be steady, since satiety or hunger had been maintained for several hours. Glycemia was monitored to check each metabolic (fasted or satiated) steady state. As shown in Table 1, glycemia was significantly higher in satiated than in fasted state and in *fa/fa* than in *fa/+* rats. The effects of feeding state and genotype on [Gluc]_ECF_ in OB and cortex were analyzed by using a three-way ANOVA with brain areas (OB and cortex), feeding state and genotype as factors. A significant effect on [Gluc]_ECF_ was observed during the initial steady state for factors “brain areas” and “genotype” (*F*_(1,24)_ = 13.6, *P* < 0.005; *F*_(1,24)_ = 21.6, *P* < 0.0001 respectively, Figure 7). [Gluc]_ECF_ was significantly higher in the OB than in the cortex, and in *fa/fa* than *fa/+* rats (SNK *post hoc* tests). No significant effect of feeding state was observed on [Gluc]_ECF_ despite differences in plasma glucose levels between satiated and fasted rats (Table 1). For each genotype, [Gluc]_ECF_ was significantly higher in OB compared to cortex (Wilcoxon test, * *P* < 0.05 for each rat genotype; in mM: Fasted: *fa/+* in the OB, 1.48 ± 0.35, in the cortex, 0.92 ± 0.33; *fa/fa* OB, 3.05 ± 0.5, cortex, 1.67 ± 0.23; Satiated: *fa/+* OB, 1.48 ± 0.25; cortex, 0.55 ± 0.09; *fa/fa* OB, 2.35 ± 0.5; cortex 1.5 ± 0.15, Figure 7). In both OB and cortex [Gluc]_ECF_ were higher in *fa/fa* compared to *fa/+* (Mann Whitney test, ^€^ *P* < 0.05 for each area brain, Figure 7).

#### During dynamic states following insulin and glucose injections

**Figure 8:**
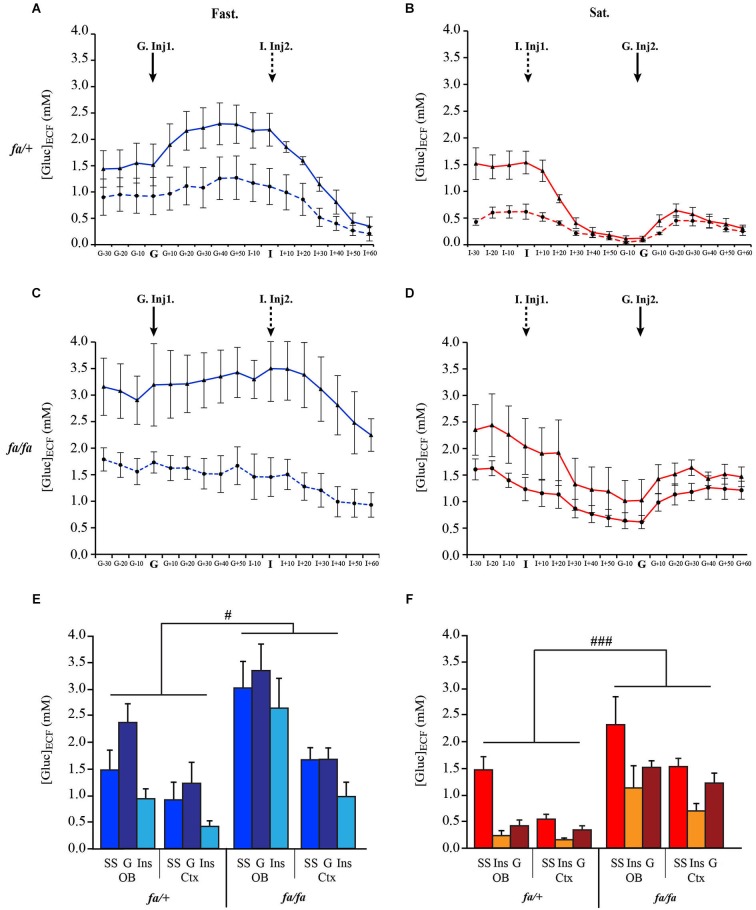
**Genotype effect on the dynamic response of [Gluc]_**ECF**_ in OB and cortex to glucose and insulin peripheral injections**. Composite figures compiled from *in vivo* real time recordings of [Gluc]_ECF_, performed simultaneously in OB (full line) and cortex (Ctx, dotted line) of lean *fa/+* **(A, B)** and obese *fa/fa* rats **(C, D)** in fasted **(A, C: Fast., blue)** and satiated **(B, D Sat., red)** states. In fasted rats, glucose was injected in first (G. Inj1), and insulin in second (I. Inj2). The order of injections was reversed in satiated rats (I-Inj1, G-Inj2). For fasted **(E)** and satiated **(F)** rats, mixed ANOVA revealed a significant effect of genotype (in fasted rat ^#^ *P* < 0.01; in satiated rats ^###^ *P* < 0.0005), conditions (not shown) and brain areas (not shown). SS: steady State; G: glucose; I: insulin.

Real time *in vivo* measurements of [Gluc]_ECF_ were recorded simultaneously from the OB and the somatosensory cortex of *fa/+* and *fa/fa* Zucker rats either fasted (Figures 8A,C respectively) or satiated (Figures 8B,D respectively). For each genotype, the two groups of rats (fasted and satiated) received two injections: one of glucose (G-Inj) and one of insulin (I-Ins). Fasted rats (Figures 8A,C) first received a glucose injection (G-Inj1) followed by an insulin injection (I-Inj2). This order was reversed for satiated rats (Figures 8B,D, I-Inj1, G-Inj2). Glucose and insulin injections induced substantial fluctuations in extracellular glucose levels, especially in the OB of *fa/+* rats. In *fa/fa* compared to *fa/+* rats, glucose injection (G-Inj) induced less pronounced variations of [Gluc]_ECF_. and insulin injection (I-Inj) induced a slower decrease of [Gluc]_ECF_. For each feeding state (satiated and fasted) a mixed ANOVA was performed with an intra-subject factor, corresponding to the three conditions (steady-state, Inj1 and Inj2) and two inter-subject factors: genotype (lean and obese) and brain area (OB and Cortex). Both in fasted (Figure 8E) and satiated (Figure 8F) rats, the statistical analysis showed a significant difference between conditions (*F*_(2,24)_ = 26.66, *P* < 0.0001; *F*_(2,24)_ = 29.28, *P* < 0.0001, respectively), genotype (*F*_(1,24)_ = 8.35, ^#^ *P* < 0.01 Figure 8E; *F*_(1,24)_ = 22.48, ^###^ *P* < 0.0005, respectively Figure 8F) and brain areas (*F*_(1,24)_ = 11.91, *P* < 0.005; *F*_(1,24)_ = 5.56, *P* < 0.05, respectively). In each feeding state (satiated and fasted), [Gluc]_ECF_ was higher in *fa/fa* than in *fa/+* rats, and in the OB than in the cortex (SNK *post hoc* tests). Moreover G-Inj increased [Gluc]_ECF_ significantly above steady-state and I-Inj. levels, and I-Inj decreased [Gluc]_ECF_ significantly below steady-state and G-Inj levels (SNK *post hoc* tests).

**Figure 9:**
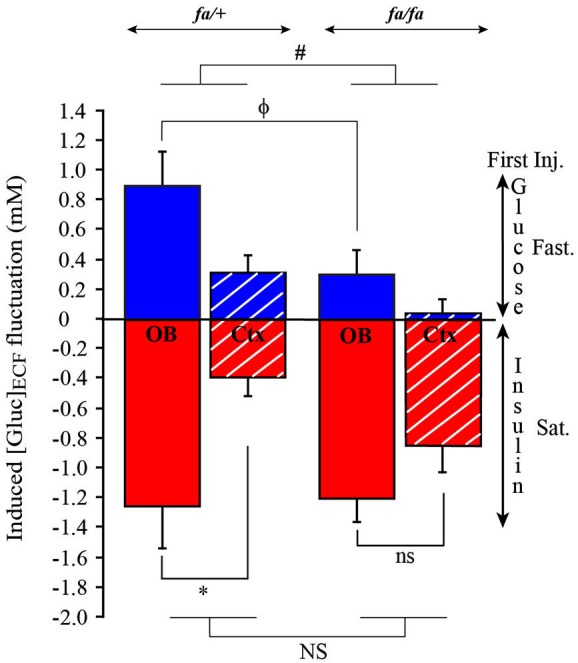
**Genotype effect on [Gluc]_**ECF**_ fluctuations induced by the first injection of either Glucose or Insulin in OB and cortex**. Bars represent the difference calculated between [Gluc]_ECF_ measured at steady state and after the first injection of glucose **(blue)** or insulin **(red)**. [Gluc]_ECF_ fluctuations were observed in OB **(full bar)** and cortex (Ctx, **dotted bar)** of lean *fa/+* **(left)** and obese *fa/fa* rats **(right)**. When glucose was injected in first in fasted rats (Fast.), a significant effect of the genotype on [Gluc]_ECF_ fluctuation was observed (ANOVA test, ^#^ *P* < 0.05; NS *P* > 0.05), [Gluc]_ECF_ fluctuation being smaller in the OB of *fa/fa* compared to *fa/+* rats (Mann Whitney, ^ϕ^ *P* < 0.05). No difference was observed in the Ctx. When insulin was injected in first in satiated rats (Sat.), only in *fa/+* rats [Gluc]_ECF_ fluctuation was significantly higher in OB than in cortex (Mann Whitney, * *P* < 0.05; ns *P* > 0.05).

Next, we calculated the difference of [Gluc]_ECF_ measurements between steady state and after the first injection (G-Inj1 or I-Inj1). This measurement corresponds to the fluctuations of [Gluc]_ECF_ shown in Figure 9. In each group (satiated and fasted rats) a two way ANOVA was performed with brain areas (OB and cortex) and genotype (*fa/+* and *fa/fa*) as factors. The ANOVA demonstrated a significant effect of brain areas in fasted (*F*_(1,12)_ = 7.03, *P* < 0.01) and satiated (*F*_(1,12)_ = 9.81, *P* < 0.01) rats. Greatest [Gluc]_ECF_ fluctuations were observed in the OB compared to the cortex (SNK *post hoc* tests). The ANOVA also demonstrated a significant difference on [Gluc]_ECF_ fluctuation between *fa/+* and *fa/fa* rats, but only in fasted rats (Figure 9, *F*_(1,12)_ = 5.1, ^#^ *P* < 0.05), the fluctuations being greatest in *fa/+* than in *fa/fa* rats. Further analysis demonstrated that this was due to fluctuations in the OB, because glucose injections had induced smallest fluctuations of [Gluc]_ECF_ in the OB of *fa/fa* rats than of *fa/+* (Mann Whitney test ^ϕ^ *P* < 0.05). In satiated *fa/+* rats only, insulin injection, had induced a greater [Gluc]_ECF_ fluctuation in the OB than in the cortex (Figure 9, Mann Whitney tests * *P* < 0.05).

## Discussion

The present study provides new information on the impact of a metabolic disorder on olfactory abilities and glucose-sensing in the olfactory system. Indeed, obese *fa/fa* Zucker rats, displayed a higher olfactory sensitivity than lean *fa/+* rats, regardless of their feeding status (fasted or satiated). In addition, the expression of glucose-sensing markers and glucose fluctuations in the OB were strikingly different between the two rat genotypes. First, SGLT1 expression was higher in the OB of obese rats compared with lean rats; while GLUT4 levels were similar for the two rat genotypes. Second, [Gluc]_ECF_ measured at steady states (fasted or satiated) was higher in *fa/fa* rats than in *fa/+* rats, with a consistently higher concentration measured in the OB compared to the cortex. Third, OB [Gluc]_ECF_ was differentially affected in *fa/fa* and *fa/+* rats when glycemia was dynamically modified by peripheral injections of glucose or insulin. Indeed, after glucose injection (acute hyperglycemia), obese rats showed a slight increase of OB [Gluc]_ECF_ (≈0.2 mM), whereas lean rats demonstrated a much higher increase (0.9 mM). Insulin injection (acute hypoglycemia) decreased [Gluc]_ECF_ similarly in the OB of the two rat strains. Differences observed in olfactory detection between obese and lean rats will be discussed in link with the modulation of glucose-sensing and of food intake.

### Obese *fa/fa* Zucker rats have higher OB insulin concentration and higher [*GLUC*]_ECF_ than lean *fa/+* rats

In addition to peripheral hyperinsulinemia and hyperglycemia, obese Zucker *fa/fa* rats displayed higher OB insulin levels than their lean counterparts. This is consistent with previous studies that reported that insulin level is highly increased in the cerebrospinal fluid of *fa/fa* compared to *fa/+* Zucker rats (Stein et al., 1983; Figlewicz et al., 1985). These results provide evidence that brain insulin is derived from circulating insulin and suggest that obese Zucker rats do not have any defect of insulin transport through the blood brain barrier (BBB). Although some early contradictory studies suggested that insulin binding was altered in the brain of obese *fa/fa* Zucker rats (Melnyk, 1987; Wilcox et al., 1989), subsequent reports convincingly demonstrated that insulin receptors (IRs) had similar expression, number, distribution, binding affinity and tyrosine kinase activity in the brain of lean and obese Zucker rats (Livingston et al., 1993; Amessou et al., 2010). Interestingly, rats carrying at least one copy of the *fa* allele (*fa/fa* and *fa/+*) present lower insulin concentration (Baskin et al., 1985) and lower insulin binding (Figlewicz et al., 1985) than non-mutated +/+ rats. Therefore, these deficits are independent of a severe obese phenotype because they are found in obese *fa/fa* and lean *fa/+* rats. Altogether, these data indicate that changes in insulin signaling are unlikely to account for the differences observed in the present study.

In both OB and cortex, [Gluc]_ECF_ was found to be higher in *fa/fa* than in *fa/+* Zucker rats. This is consistent with the higher brain glucose uptake observed in obese rats compared to lean controls (Liistro et al., 2010). It is interesting to note that a higher central glucose availability is associated with a lower local cerebral glucose utilization (Doyle et al., 1993), especially in brain areas implicated in the neuroendocrine regulation of food intake and in odor-taste perception, such as the hypothalamus and the OB (Marfaing-Jallat et al., 1992). Moreover, in both rat genotypes, [Gluc]_ECF_ at steady states was found to be higher in the OB than in the cortex and this is consistent with the compartmentalization of [Gluc]_ECF_ according to the brain area studied and to the level of neural activity (McNay and Gold, 1999; McNay et al., 2001). Work from the group of Magistretti has demonstrated that neuronal activity and glucose metabolism are tied together (Magistretti et al., 1999). Indeed, OB presents a higher functional activity than the somatosensory cortex (Yang et al., 1998). Moreover, the OB glomerular layer not only has remarkably high glucose consumption rates (Nawroth et al., 2007) but also possesses very high capillary network density (Chaigneau et al., 2007) combined with a distinct microvasculature (Yang et al., 1998) and a high permeability of the BBB (Ueno et al., 1996). Together these hallmarks suggest that OB not only elicits high-energy demands but could also be a glucose-sensing brain area.

### Obese *fa/fa* Zucker rats have a higher expression of Glucose-sensing molecular markers in the OB than lean *fa*/+ rats

In the OB of both rat genotypes, the molecular markers of glucose-sensing GLUT4 and SGLT1 were detected in different layers of the OB. GLUT4 immunostaining was observed in glomeruli and in some mitral cells, while SGLT1 was mainly observed in the iEPL. This distribution is similar to that shown by *in situ* hybridization in Allen Brain Atlas (Allen Institute for Brain Science, 2010). This distinct regional location of GLUT4 and SGLT1 support the idea that these two families of glucose transporters play different roles in neuronal network processing as it has been previously reported in other brain areas (Yu et al., 2010, 2013).

Obesity did not modulate GLUT4 expression in the OB, since obese *fa/fa* and lean *fa/+* rats presented similar GLUT4 localization and level of expression. However, obesity changed SGLT1 expression, which was higher in satiated *fa/fa* than in satiated *fa/+*. Concerning GLUT4, our results are in agreement with previous work performed on hyperinsulinemic-hyperglycemic db/db mice, which showed that GLUT4 protein levels were unchanged in the cortex and the OB (Vannucci et al., 1998). Accordingly, GLUT4 mRNA levels did not change in the cortex of hyperinsulinemic *fa/fa* Zucker rats (Alquier et al., 2006). Winocur and collaborators suggested that the stability of GLUT4 expression is related to the insulin resistance of *fa/fa* Zucker rats (Winocur et al., 2005), because GLUT4 translocation to the plasma membrane can no longer be triggered by insulin signaling as it is in a normal metabolic context (McEwen and Reagan, 2004). Indeed, in the hippocampus of Zucker rats, GLUT4 association to the plasma membrane was significantly reduced although total GLUT4 protein expression was not affected (Winocur et al., 2005). In order to confirm this hypothesis, it will be interesting to further study and compare GLUT4 subcellular localization in the OB of the two genotypes of Zucker rats. Concerning SGLT1, the up-regulation observed in the OB of *fa/fa* rats is consistent with previous studies performed on peripheral tissues in obese, hyperinsulinemic and diabetic rodent models as well in human (Morton and Hanson, 1984; Ferraris and Vinnakota, 1995; Dyer et al., 2002; Osswald et al., 2005; Tabatabai et al., 2009). In central nervous system, during pathological conditions such as ischemia or epileptic seizure which induce an over-consumption of glucose (Poppe et al., 1997; Elfeber et al., 2004) SGLT1 was up-regulated (Yu et al., 2010, 2013). Up-regulation of SGLT1 is proposed to compensate for impairment in GLUTs function (Yu et al., 2013). Thus, in the context of insulin resistance, the deficiency in GLUT4 translocation could explain the SGLT1 up-regulation.

### Fasted rats have a better olfactory sensitivity than satiated rats, regardless of the genotype

Olfactory sensitivity of both *fa/+* and *fa/fa* Zucker rats is modulated by the feeding states. They have lower olfactory detection and detect the aversive odorant more quickly in the fasted state than in the satiated state, as we previously observed with normal-weight Wistar rats (Aimé et al., 2007). Olfactory sensitivity is modulated by a number of peripheral and central signals involved in the regulation of energy balance such as leptin, orexin A, ghrelin and insulin (Julliard et al., 2007; Tong et al., 2011; Aimé et al., 2012; Palouzier-Paulignan et al., 2012). The OB is the target of orexinergic fibers originating from the lateral hypothalamic nucleus (Peyron et al., 1998). In addition, the olfactory system expresses, among others, receptors for orexin A, ghrelin, leptin, insulin, NPY and CCK (for review, see Palouzier-Paulignan et al., 2012). Our results suggest that although many of these signals are altered in the obese Zucker *fa/fa* rats, the redundancy of orexigenic and anorectic molecules acting on the olfactory system can ultimately maintain the modulation of olfactory sensitivity by the feeding state. For instance, we found that the OB insulin content in both *fa/fa* and *fa/+* Zucker was modulated by the feeding states, with satiated animals showing a higher OB insulin level than fasted animals. Interestingly, we have previously demonstrated in Wistar rats that such fluctuations of OB insulin levels are sufficient to decrease the olfactory sensitivity of fasted animals to the level of satiated ones (Aimé et al., 2012). Changes of insulin levels in OB and in plasma were correlated confirming that the OB is highly sensitive to fluctuations in circulating insulin levels. This result is consistent with the general agreement that OB is the brain region containing the highest level of IRs (Hill et al., 1986; Unger et al., 1989; Marks et al., 1990). This receptor allows pancreatic insulin to enter the brain across brain capillaries (Schwartz et al., 1990; Banks et al., 1997; Woods et al., 2003; Banks, 2004). At the entire brain level, the rate of insulin entrance is regulated by several physiological factors, including the feeding state (Woods et al., 2003; Banks, 2004). When animals are fasted, the ability of insulin to cross the BBB is reduced, leading to a positive correlation between blood and cerebrospinal fluid insulin levels (Strubbe et al., 1988). Although one report suggested that OB insulin binding is modulated by the feeding state and reduced by chronic fasting (Marks and Eastman, 1989), we have recently shown that the OB IR expression is not modulated by the feeding state (Aimé et al., 2012). Consistent with the latter, nutrient availability does not modulate IR levels in brain regions involved in energy homeostasis regulation (Bowlby et al., 1997). Together, these data indicate that insulin signaling in the OB is dependent on the fluctuations of peripheral insulin levels, and participate in the modulation of synaptic transmission of odor-related stimuli by the feeding states.

Interestingly, both genotypes showed similar OB [Gluc]_ECF_ in steady fasted or satiated states although glycemia was much higher in the satiated state. This is consistent with our previous observation in Wistar rats (data not shown) and could be due to adjustments of the glucose transport capacity at the BBB in response to brain metabolic rate and glucose availability (for review see Bradbury, 1993; Leybaert, 2005; Banks, 2006). Three mechanisms have been proposed to explain the modulation of glucose transport across the BBB (see for review Leybaert, 2005) an increase in: (i) the number of GLUT1; (ii) the intrinsic activity of the GLUT1; and/or (iii) the glucose concentration gradient over the barrier, thereby stimulating the driving force for glucose movement. It is possible that in metabolic disorders, these three mechanisms are not altered all together or at the same time. In the present study, the OB [Gluc]_ECF_ stability observed in *fa/fa* rats could be the result of the modulation of either GLUT1 expression or intrinsic activity at the BBB. In future experiments, it will be interesting to study more precisely these different mechanisms of glucose transport through the BBB during the different steps of feeding state in the OB of *fa/fa* and *fa*/+ Zucker rats.

### Obese *fa/fa* Zucker rats have a higher olfactory sensitivity than lean *fa*/+ rats

In the present study, Zucker *fa/fa* rats demonstrated a better olfactory sensitivity than Zucker *fa/+* rats. This finding is supported by several recent studies. Thanos and collaborators demonstrated that *fa/fa* Zucker rats displayed altered brain metabolic responses to food olfactory stimuli in several brain regions (Thanos et al., 2008). The same group also demonstrated that olfactory cues for a high-fat food stimulus elicit heightened behavioral responses in the obese Zucker *fa/fa* rat compared to lean Zucker *fa/+* rats (Thanos et al., 2013). Interestingly, obese ob/ob mice (unable to produce leptin) and db/db mice (carrying a random mutation in the leptin receptor gene) are able to locate a hidden food reward based on olfactory cues, much faster than control mice. Several metabolic dysfunctions could explain the heightened olfactory perception of obese rats. As previously proposed, leptin is likely to mediate these responses (Thanos et al., 2013). Indeed, *fa/fa* rats are insensitive to leptin, an anorectic signal known to decrease olfactory sensitivity (Julliard et al., 2007; Aimé et al., 2012). The *fa* mutation of the Zucker rat (*fa/fa*) affects the leptin receptor gene and prevents the long form of the receptor (Ob-Rb) from being expressed (Chua et al., 1996). The Ob-Rb form of the receptor is expressed in the brain (Mercer et al., 1996; Fei et al., 1997) and is present at different levels of the olfactory system, including the OB (Shioda et al., 1998; Getchell et al., 2006; Baly et al., 2007; Prud’homme et al., 2009). Previous reports have demonstrated that leptin is able to modulate olfactory behavior as well as neuronal activity (Getchell et al., 2006; Julliard et al., 2007; Prud’homme et al., 2009; Savigner et al., 2009). Indeed, ICV administration of leptin decreases the expression of the activation marker c-fos in mitral and granular OB neurons (Prud’homme et al., 2009). Consistent with these studies, in a previous report, we found that ICV administration of leptin decreases the olfactory sensitivity of fasted animals to the level of satiated ones (Julliard et al., 2007). Here, we demonstrated that Zucker *fa/fa* rat, expressing a non-functional form of the leptin receptor, display a better olfactory sensitivity than the Zucker *fa/+* rats, consistent with the aforementioned reports showing that leptin is responsible for a decrease in olfactory abilities.

Other metabolic dysfunctions could contribute to increase the olfactory perception of obese rats. The level of orexigenic peptides such as neuropeptide Y, ghrelin and orexin A is upregulated in the hypothalamus of obese Zucker rats (Beck et al., 1990, 2003; Sanacora et al., 1990; Mondal et al., 1999) and ghrelin and orexin A are known to increase olfactory sensitivity (Julliard et al., 2007; Tong et al., 2011). Zucker *fa/fa* rats are also insensitive to the anorectic action of CCK (McLaughlin and Baile, 1980); and the expression level of proopiomelanocortin (POMC), the precursor of the anorectic neuroptide αMSH, is decreased in the arcuate nucleus of *fa/fa* rats (Yamamoto et al., 2002). Although peripheral insulin injection induced similar fluctuations of OB [Gluc]_ECF_ in *fa/fa* and *fa/+* Zucker rats, peripheral glucose injection affected OB [Gluc]_ECF_ differentially in lean and obese Zucker rats. A peripheral glucose injection induced a greater increase of OB [Gluc]_ECF_ in the OB of Zucker *fa/+* rats, compared to Zucker *fa/fa* rats. This result is supported by a recent report that found no significant difference of brain glucose levels in obese Zucker rats after glucose injection compared to fasting brain glucose levels (Liistro et al., 2010). The brain of obese animals is chronically overexposed to glucose (even during fasting), as a consequence, it seems no more able to respond to physiologic glucose fluctuations. Importantly, the alteration of the molecular markers of glucose-sensing (up-regulation of SGLT1 expression and a probable alteration of GLUT4 trafficking) could also participate to the differences of olfactory detection observed between the two genotypes. Indeed, both SGLT1 and GLUT4 have been shown to be involved in neuronal glucose-sensing (McEwen and Reagan, 2004; O’Malley et al., 2006; Yu et al., 2013). This sensitivity for glucose is observed in specialized neurons located in numerous brain regions involved directly or indirectly in homeostasis control (for review see Routh, 2010; Levin et al., 2011), including the OB (Tucker et al., 2010, 2013). In the OB, the mitral cells have recently been demonstrated to be glucose sensitive (Tucker et al., 2010, 2013) therefore glucose signaling could participate to the modulation of the olfactory sensitivity. Glucose-sensing neurons adapt their mean firing rate to the local fluctuations of extracellular glucose levels (González et al., 2008; McCrimmon, 2008). It is well-known that glucose-sensing is altered in the hypothalamus of obese animals (Rowe et al., 1996; Spanswick et al., 1997, 2000), and alteration of glucose-sensing in the olfactory system could play a role in the modulation of olfactory sensitivity observed in *fa/fa* rats. Altogether, Zucker *fa/fa* rats overexpress orexigenic peptides known to increase olfactory sensitivity (Julliard et al., 2007; Tong et al., 2011) and are insensitive to anorectic signals known to decrease olfactory sensitivity (Julliard et al., 2007; Aimé et al., 2012) and they present an altered glucose-sensing in the OB. This imbalance in nutrient sensing, in orexigenic and anorectic signals is likely to account for the increase of olfactory sensitivity demonstrated here by the Zucker *fa/fa* rats. However in the present study, it remains difficult to decipher the respective role of obesity and of glucose-sensing alteration related to hyperglycemia on olfactory sensitivity. In order to go further, it will be intriguing to study olfactory sensitivity and expression of glucose-sensing genes on female Zucker rats because numerous gender-related metabolic differences exist in peripheral (Clark et al., 1983; Corsetti et al., 2000; Gustavsson et al., 2011) as well as in central tissues (Bogacka et al., 2004). Indeed, females develop obesity and insulin resistance but remain normoglycemic (Clark et al., 1983; Corsetti et al., 2000) and express differently glucose-sensing genes in hypothalamus compared to males (Bogacka et al., 2004).

## Conclusion

The whole of these data indicate that obese Zucker *fa/fa* rats demonstrate marked glucose intolerance and an alteration in the expression of glucose-sensing markers in the OB. These newly found impairments, along with the well-described multiple metabolic dysfunctions of obese Zucker *fa/fa* rats modulate the processing of olfactory information and contribute to increase the olfactory sensitivity. Ultimately, changes in olfactory sensitivity participate in the hyperphagia and food-related disorder characteristic of the Zucker *fa/fa* rat.

## Authors contributions

All experiments were performed in the Centre de Recherche en Neurosciences de Lyon (CRNL). Inserm U1028-CNRS 5292- UCBL1, Team—Olfaction: From Coding to Memory, Lyon, France. Pascaline Aimé and A. Karyn Julliard were responsible for the conception and design of the experiments; Pascaline Aimé, Rita Salem, Caroline Romestaing, Dolly Al Koborssy and Claude Duchamp were responsible for the collection of the data; Pascaline Aimé, Brigitte Palouzier-Paulignan, Rita Salem and A. Karyn Julliard were responsible for data analysis and interpretation; and Samuel Garcia designed the software used in analysis. Pascaline Aimé, Brigitte Palouzier-Paulignan, Claude Duchamp and A. Karyn Julliard drafted the article and revised it critically for important intellectual content. All authors approved the final version of the manuscript.

## Conflict of interest statement

The authors declare that the research was conducted in the absence of any commercial or financial relationships that could be construed as a potential conflict of interest.
